# Researching effective approaches to cleaning in hospitals: protocol of the REACH study, a multi-site stepped-wedge randomised trial

**DOI:** 10.1186/s13012-016-0406-6

**Published:** 2016-03-24

**Authors:** Lisa Hall, Alison Farrington, Brett G. Mitchell, Adrian G. Barnett, Kate Halton, Michelle Allen, Katie Page, Anne Gardner, Sally Havers, Emily Bailey, Stephanie J. Dancer, Thomas V. Riley, Christian A. Gericke, David L. Paterson, Nicholas Graves

**Affiliations:** 1 Institute of Health and Biomedical Innovation, Queensland University of Technology, GPO Box 2434, Brisbane, QLD 4001 Australia; 2 Faculty of Nursing and Health, Avondale College, 185 Fox Valley Road, Wahroonga, NSW 2076 Australia; 3 School of Nursing, Midwifery and Paramedicine, Australian Catholic University, PO Box 256, Dickson, ACT 2062 Australia; 4 Department of Microbiology, Hairmyres Hospital, Eaglesham Rd, East Kilbride, G75 8RG UK; 5 School of Pathology and Laboratory Medicine, University of Western Australia, 35 Stirling Hwy, Crawley, WA 6009 Australia; 6 School of Public Health, University of Queensland, Herston, QLD 4006 Australia; 7 Wesley Medical Research, Wesley Hospital, PO Box 499, Toowong, QLD 4066 Australia; 8 University of Queensland Centre for Clinical Research, Royal Brisbane and Women’s Hospital, Herston, QLD 4029 Australia

**Keywords:** Hospital cleaning, Cleaning bundle, Cost-effectiveness, Healthcare-associated infection, iPARIHS

## Abstract

**Background:**

The Researching Effective Approaches to Cleaning in Hospitals (REACH) study will generate evidence about the effectiveness and cost-effectiveness of a novel cleaning initiative that aims to improve the environmental cleanliness of hospitals. The initiative is an environmental cleaning bundle, with five interdependent, evidence-based components (training, technique, product, audit and communication) implemented with environmental services staff to enhance hospital cleaning practices.

**Methods/design:**

The REACH study will use a stepped-wedge randomised controlled design to test the study intervention, an environmental cleaning bundle, in 11 Australian hospitals. All trial hospitals will receive the intervention and act as their own control, with analysis undertaken of the change within each hospital based on data collected in the control and intervention periods. Each site will be randomised to one of the 11 intervention timings with staggered commencement dates in 2016 and an intervention period between 20 and 50 weeks. All sites complete the trial at the same time in 2017. The inclusion criteria allow for a purposive sample of both public and private hospitals that have higher-risk patient populations for healthcare-associated infections (HAIs). The primary outcome (objective one) is the monthly number of *Staphylococcus aureus* bacteraemias (SABs), *Clostridium difficile* infections (CDIs) and vancomycin resistant enterococci (VRE) infections, per 10,000 bed days. Secondary outcomes for objective one include the thoroughness of hospital cleaning assessed using fluorescent marker technology, the bio-burden of frequent touch surfaces post cleaning and changes in staff knowledge and attitudes about environmental cleaning. A cost-effectiveness analysis will determine the second key outcome (objective two): the incremental cost-effectiveness ratio from implementation of the cleaning bundle.

The study uses the integrated Promoting Action on Research Implementation in Health Services (iPARIHS) framework to support the tailored implementation of the environmental cleaning bundle in each hospital.

**Discussion:**

Evidence from the REACH trial will contribute to future policy and practice guidelines about hospital environmental cleaning. It will be used by healthcare leaders and clinicians to inform decision-making and implementation of best-practice infection prevention strategies to reduce HAIs in hospitals.

**Trial registration:**

Australia New Zealand Clinical Trial Registry ACTRN12615000325505

## Background

Healthcare-associated infections (HAIs) are a major cause of avoidable costs, morbidity and deaths among hospital patients [1]. In Australia, 200,000 cases of HAI arise each year and 1.9 million hospital bed days are diverted to treat them [1]. Reducing HAIs requires successful implementation of multiple evidence-based approaches [2].

While reasonable knowledge exists on many aspects of HAI prevention and control, good quality data on the effectiveness of hospital environmental cleaning programmes are limited. The healthcare environment plays a key role in the transmission of HAIs [3–5]. Environmental cleaning and evaluating cleanliness is therefore a critical component of HAI prevention. Most research examining the association between cleaning and reduction of infections has been based on a single ward or hospital using quasi-experimental designs [6]. There is little published evidence on cleaning relating to intervention cost, cost-effectiveness, feasibility and acceptability. Decision makers need more rigorous evidence from randomised controlled trials in real-world settings [7].

Environmental cleaning in hospitals is a complex process, and implementing and sustaining effective cleaning programmes is challenging [4, 8]. A review of hospital cleaning in Australian hospitals found great heterogeneity in cleaning practices between hospitals even though there are detailed cleaning guidelines [9, 10].

### Using bundles to improve implementation of complex interventions

A bundle is a set of evidence-based practices that when performed collectively and reliably have a proven ability to improve patient outcomes [11]. Using a bundle is a structured way to introduce a number of interventions concurrently, improving implementation and impact on processes of care.

An environmental cleaning initiative that combines multiple evidence-based interventions within a ‘cleaning bundle’ has the potential to improve the effectiveness of hospital cleaning by including strategies to educate, provide feedback and empower healthcare workers. A multi-site trial of an environmental cleaning bundle will allow a change in cleaning policy and practice to be evaluated in real-world settings to determine efficacy and cost-effectiveness. This trial will provide evidence that can be used by clinicians and decision makers to inform future environmental cleaning and infection prevention in hospitals. This trial is called Researching Effective Approaches to Cleaning in Hospitals (REACH).

### Trial objectives

#### Objective 1

The first objective is to evaluate the effectiveness of an environmental cleaning bundle to reduce HAIs in Australian hospitals.

#### Objective 2

The second objective is to estimate the cost-effectiveness of a decision to adopt the environmental cleaning bundle for Australian hospitals.

## Methods/design

### Study design

#### Stepped-wedge design

**Fig. 1:**
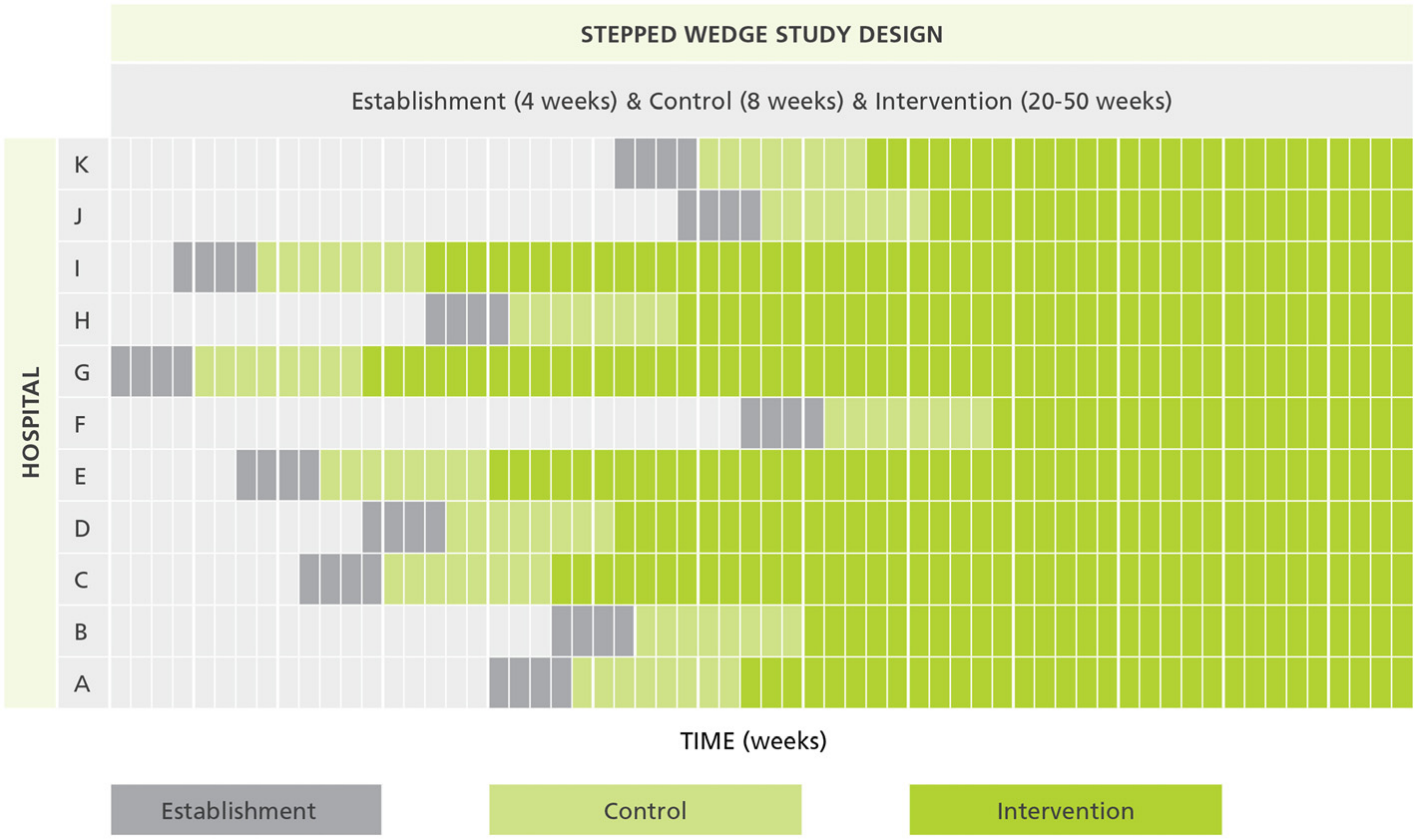
Example stepped-wedge trial timings over 62 weeks in 11 Australian hospitals

This is a randomised controlled trial using a cross-sectional stepped-wedge random allocation [12]. There will be sequential roll-out of an environmental cleaning bundle intervention to 11 Australian public and private hospitals over 62 weeks. Example trial timings are shown in Fig. 1.

All hospitals will receive the intervention and act as their own control, with analysis undertaken of the change within each hospital based on data collected in the control and intervention phases. The stepped-wedge design, with staggered commencement timings and intervention length (20–50 weeks), supports feasibility while maintaining the rigour of the study. This design will allow research staff to work with individual hospitals as they change-over, maximising consistency of implementation of the cleaning bundle and avoiding managing change at 11 sites across Australia simultaneously. This design also avoids having control hospitals that get no intervention, which could make it difficult to recruit hospitals, and ensures equity. Each hospital acting as their own control avoids inconsistencies in comparing infection control programmes across hospitals and jurisdictions.

#### Implementation framework

The project will use the integrated Promoting Action on Research Implementation in Health Services (iPARIHS) [13] framework to promote the successful implementation of the evidence-based intervention at trial sites [14]. This framework has key constructs of innovation (e.g. the cleaning bundle), recipients (e.g. the environmental services staff) and context (e.g. hospital characteristics) that are underpinned by a fourth construct of facilitation (e.g. engagement strategies) [13].

### Study population

Eleven large acute public and private Australian hospitals that fulfil the inclusion criteria and agree to participate will be enrolled in the study. These criteria are that each hospital:

1. Has an intensive care unit (ICU) accredited for advanced clinician training by the College of Intensive Care Medicine of Australia & New Zealand [15]
2. Is classified by the National Health Performance Authority (NHPA) as a major hospital (public hospital) or has over 200 in-patient beds (private hospital) [16]
3. Has an established HAI surveillance programme that collects data on *Staphylococcus aureus* bacteraemia (SAB) infections, *Clostridium difficile* infections (CDIs) and vancomycin resistant enterococci (VRE) infections

#### Recruitment

The study team will list all eligible sites then order the list to ensure (i) a representation of both private and public hospitals and (ii) representation from at least four Australian states and territories. The recruitment process will purposively select and approach eligible hospitals to optimise the feasibility and practicality of completing the trial.

### The intervention

#### Developing and piloting

In 2014, Allen and colleagues, from the National Health and Medical Research Council (NHMRC) Centre of Research Excellence in Reducing Healthcare Associated Infections (CRE-RHAI), conducted a single-site pilot of an environmental cleaning behavioural change bundle. The bundle components were agreed, following a review of the evidence and feasibility, by an expert panel and piloted successfully at a large acute hospital in Queensland in 2014 [17]. The pilot study demonstrated that the intervention is acceptable to hospital staff, that the outcomes can be reliably measured and that the cleaning bundle is feasible [17].

#### Bundle components

The multimodal intervention has five interdependent components, shown in Table 1, and will be delivered as a hospital-wide intervention. For practical reasons the cleaning audits will sample a percentage of wards only. The other bundle components (training, technique, product and communication) will be implemented across the whole hospital to allow for staff relocation to and from sample wards.

**Table 1:** Environmental cleaning bundle

Bundle component	Key activities
Training	- Tailored training activities with environmental services staff at the commencement of the intervention phase, as part of induction for new cleaning staff, and as required throughout the intervention phase - Content to reflect the trial site context and cleaning roles and responsibilities
Technique	Attention to cleaning technique, including: - A defined and consistent cleaning sequence - A focus on cleaning high risk frequent touch points - The use of sufficient pressure and movement - Adherence to manufacturers’ instructions for product use
Product	- Disinfectant minimally used for all discharge cleans and for daily cleans of high risk/precautions rooms - Point of care wipes used for medical equipment
Audit	- Audit activities across the trial site using ultraviolet (UV) marker technology (all trial sites) and adenosine tri-phosphate (ATP) luminosity (3 trial sites) - Regular audit feedback to cleaning staff - Summarised audit results provided to clinical governance committees
Communication	- Promotion of a team approach - Daily contact between cleaners and ward leaders or managers - Cleaners represented on relevant clinical governance committees

The bundle components reflect the current evidence concerning hospital cleaning and HAIs about the positive impact of audit activities [18, 19], educational interventions [20, 21], clearly defined cleaning roles and responsibilities [22] and correct product use along with daily cleaning of the frequent touch points. Product and technique components align with current Australian Guidelines [23]. Communication, particularly a positive feedback loop in association with audit [20, 22], and creation of a ‘culture of hygiene’ [24], will be emphasised throughout the intervention phase.

#### Implementation

The study team will use the iPARIHS framework to support effective implementation of the cleaning bundle intervention at each site [13, 14]. A tailored intervention should optimise bundle implementation and compliance. The tailoring will not compromise the integrity of the individual components and the ‘sum of the whole’ of the bundle.

Initially, we will map the hospital characteristics and context (e.g. staffing, size), infection prevention policies and practices (e.g. antimicrobial stewardship, antibiotic use, screening) and conduct surveys. This will provide the information to develop a tailored implementation strategy for each site, based on behavioural change and adult learning principles, current environmental cleaning practices and other contextual factors.

Throughout the trial, we will monitor these characteristics and other relevant activities (e.g. cleaning staff changes, policy changes) within the hospital. Ongoing reviews of contextual information throughout the intervention at each site will assist with trial site comparisons, replication and scalability and knowledge translation.

### Randomisation

Hospitals will be randomly allocated to intervention timing once 11 sites have been enrolled. One of the chief investigators (AGB, statistician) will be responsible for computer generation of the allocation times and the allocation of hospital identifiers. Hospitals will be informed of their intervention start date 12 weeks before the intervention commences. This 12-week period will include 4 weeks of establishment/engagement activities (‘establishment phase’) and 8 weeks of baseline data collection (‘control phase’). Hospitals and research sites will not be blinded because it is not possible to blind the cleaning staff to the intervention.

### Outcomes and data collection: objective 1: effectiveness of the environmental cleaning bundle

Objective 1 outcomes and measures are shown in Table 2.

**Table 2:** Objective 1 outcomes and measures

Outcome	Measure	Method
Primary outcome		
Healthcare-associated infection (HAI) rates in each trial site	Numbers of *Staphylococcus aureus* bacteraemia (SAB), *Clostridium difficile* infection (CDI) and clinical isolates of vancomycin resistant enterococci (VRE) and monthly rates per 10,000 occupied bed days	Datasets include all patients across the hospital with a HAI, and data on occupied bed days Collated from: - Hospital infection data and reports for ongoing surveillance - Hospital occupied bed days reports
Secondary outcome		
Hospital cleaning performance—thoroughness of hospital cleaning in each trial site	Number and percentage of ultraviolet (UV) gel dots removed completely following cleaning	DAZO® Fluorescent Marking Gel dots applied to frequent touch sites around two patient cubicle and bathroom areas in the intensive care unit (ICU) and the highest risk wards
Other outcomes—study specific data set		
Bio-burden of frequent touch surfaces post cleaning	Levels of organic matter in relative light units (RLUs) post cleaning in three hospitals	Adenosine tri-phosphate (ATP) bioluminescence measurements
Other outcomes—study specific data set		
Changes in staff knowledge and attitudes around environmental cleaning	Pre-intervention educational needs. Pre- and post-intervention knowledge and attitudes about environmental cleaning and job roles	Environmental services staff questionnaires and discussion groups
Other outcomes—existing data set		
Changes in rates of screening and clinical isolates	Numbers of positive clinical isolates of other multi-resistant organisms per 1000 patient days at risk	Collated from hospital infection data and reports, where available for ongoing surveillance and supplied to project team by trial site team
Other outcomes—existing data set		
Changes in patients’ perception of hospital cleanliness	Patients’ perceptions of cleanliness	Collated from existing hospital-based survey data, where available and supplied to project team by trial site team

#### Primary outcome—HAI rates

Whole hospital infection rates will be used as the primary outcome. This reflects the frequent movement of staff, patients and cleaners across hospitals wards and locations.

Hospitals will collect monthly data and then submit it at least bi-monthly to the project team. The numbers of SABs, CDIs and clinical isolates of VRE will be monitored, and rates calculated using algorithms based on nationally agreed definitions, as published by the Australian Commission on Safety and Quality in Health Care (ACSQHC) [25].

#### Secondary outcome—thoroughness of routine hospital cleaning

Thoroughness of hospital cleaning will be measured using the DAZO® Fluorescent marking gel and ultraviolet (UV) light system that dries on surfaces following application and resists dry abrasion but is removed with standard cleaning. A trial site staff member trained by the study team will apply the gel dots on frequently touched points in the patient cubicles as per the Centers for Disease Prevention and Control Environmental Cleaning Checklist [26]. On each audit occasion, dots will be placed in two different beds and bathroom areas in the ICU and in at least 50 % of the hospital trial site wards. Wards will be prioritised according to NHPA risk factors for patient vulnerability [16]. Cleaning staff will be aware of the frequently touched points but will not be aware of where the dots are actually applied or the timing of audits.

After cleaning, the dot locations will be checked using the UV light pen, which will show the extent of removal of the dot by cleaning. All results will be entered directly into a web-based application on a mobile device at the time of data collection. Cleaning audits will take place in the control and intervention periods. Feedback mechanisms for staff will be implemented as part of the bundle during the intervention period.

#### Other outcomes

##### Bio-burden of frequent touch surfaces

We will routinely assess bio-burden on a selection of high touch surfaces using adenosine tri-phosphate (ATP) luminosity following cleaning in three of the 11 hospitals, using a validated sampling process [27].

##### Knowledge and attitudes of environmental cleaning staff

The knowledge and attitudes of environmental cleaning staff will be surveyed pre- and post-intervention and will serve two purposes. First, it will provide an understanding of baseline hospital context, including knowledge, attitudes and practices of environmental services staff, which will be used to tailor the educational component of the cleaning bundle. Second, it will allow a comparison of knowledge and attitudes about environmental cleaning between the pre- and post-intervention phases. This is particularly important to gauge whether the bundle has impacted not only upon behaviour but also on attitudes and knowledge, which are the longer-term drivers of behavioural outcomes.

##### Other surveillance data

In areas of the hospital where patient screening for multi-resistant organisms is a standard practice (such as in ICU) and consistently undertaken during the study period, data on screening isolates, via nasal swabs for Methicillin-resistant *Staphylococcus aureus* (MRSA) and stool or peri-rectal swabs for VRE per 1000 patient days, will also be collated.

##### Patient satisfaction surveys

These will only be used if an existing survey tool and process is used by the hospital and if patient survey collation/report periods align with the control and intervention timings at that trial site.

#### Power calculation

Eleven hospitals with a pre-intervention infection rate (a combination of SAB, CDI and VRE infections) of 5 per 10,000 patient days will give us 86 % power to detect a 20 % post-intervention reduction in infection risk. This is based on a two-sided 5 % significance level, a within-hospital correlation in infection rates of 0.3 and the intervention timings shown in Fig. 1. The pre-intervention infection rate and within-hospital correlation were estimated using a dataset of over two million hospital admissions and infection data from nine Queensland hospitals [28]. The stepped-wedge sample size formula is from Hussey and Hughes [29]. We assumed no delay in the treatment effect for the sample size calculation, that is, rates decline as soon as the intervention begins.

To detect an increase in the secondary outcome (thoroughness of cleaning) from 82 to 87 % due to the intervention with a 92 % power requires only five hospitals with 40 observations per hospital per 4 weeks. This is based on the pilot data and recent studies [17, 22, 30, 31]. This secondary hypothesis requires far fewer hospitals because the outcome of cleaning failure is likely to be more common than HAIs (primary outcome), which are relatively rare.

#### Analysis

##### Infection rates

Infection rates will be analysed using Poisson regression with the infection counts as the dependent variable and the weekly number of patient days as the denominator. The key independent variable will be the intervention (yes/no). The intervention variable will switch from “no” to “yes” after the first full month of the intervention. The model will include a linear term for calendar time to control for any long-term pattern in infection rates. It will be a generalised linear mixed model (GLMM) with a random intercept for each hospital [29].

The characteristics of the hospital (e.g. size) will not be independent variables as these should remain roughly constant throughout the study. We will test the residuals of the model to look for autocorrelation over time and will check for influential observations. In a sensitivity analysis we will consider the possibility of a delayed intervention effect of up to one month. This is plausible because it may take time for the intervention to break the cycle of infection transmission.

The primary outcome will be a fixed effect meta-analysis of the estimated change in infection rates across the three infection types. We will use a forest plot to visually examine the variation in the change in infection rates. The combined mean will be considered statistically significant if the 95 % confidence limit does not include zero. The meta-analytic approach avoids using a combined infection rate, as this is questionable given the different drivers of infection. However, it will still give an overall estimate of the interventions impact on HAIs.

##### Thoroughness of cleaning

Success or failure of environmental cleaning as measured by the removal of invisible gel dots will be examined using a GLMM with a binomial response (clean/not clean). This will model the probability of success as the number of successfully clean sites divided by the total number of sites marked.

The independent variables will be the intervention (yes/no) and the dot location (e.g., ICU, toilet). The model will include a random intercept for each hospital, which focuses the analysis on within-ward change by removing each wards underlying success rate. We will test two models with independent variables of:

1. A simple binary intervention (yes/no)
2. A linear intervention using the time since intervention (0, 1, 2, etc.)

The linear model allows for a gradual improvement in success rates. We will compare the fit of the two models using the Akaike Information Criterion [32].

##### Bio-burden of high touch surfaces

The total amount of ATP, both microbial and non-microbial, will be quantified and expressed as relative light units. This will be correlated with the secondary outcome data (thoroughness of cleaning).

##### Changes in staff knowledge and attitudes

Several pre-existing scales from the literature will be used to assess both the attitudes and knowledge of environmental cleaning staff [33–36]. Paired *t* tests (or Wilcoxon signed-rank tests) will be used to analyse the changes in attitudes between the pre- and post-intervention phases. Change in knowledge will be assessed as the difference in the proportion of correct responses at time 1 versus time 2, tested using McNemar’s test. General perceived organisational support will also be assessed in a short eight-item measure [37].

##### Other surveillance data

The changes in rates of positive screening and clinical isolates will be examined as per the primary outcome.

##### Changes in patient perceptions of hospital cleanliness

As there are likely to be differences in the questions used between hospitals, we will estimate the change within each hospital and then combine these using a meta-analysis. To provide estimates on a common scale, we will look at the percentage change from baseline.

### Outcomes and data collection: objective 2: cost-effectiveness of the environmental cleaning bundle

Objective 2 outcomes and data measures and processes are shown in Table 3.

**Table 3:** Objective 2 outcomes and data sources

Outcomes	Measures		Methods
Primary outcome: Incremental cost-effectiveness ratio from adoption of the cleaning bundle presented as cost per quality-adjusted life year (QALY) gained (calculated from secondary outcomes)			
Secondary outcomes:			
1. Changes in costs associated with implementing the bundle		Frequency and value of resources used in implementing the bundle (costs incurred)	Hospital specific items collated from existing hospital-based data sets and supplied to project team by trial site team for analysis. Centralised resources recorded by project team. Valued in 2016 Australian dollars (AUD)
		Cost of infection in terms of treatment costs, diagnosis costs and bed days saved (potential cost savings)	Calculated using estimates from the literature about the attributable cost of infection. Valued in 2016 AUD
2. Changes in QALYs associated with implementing the bundle		Opportunity cost of infection in terms of: 1. Deaths 2. QALYs	Calculated using estimates from the published literature on attributable mortality and morbidity for infection

The perspective used to evaluate the cost-effectiveness of the environmental cleaning bundle will be that of the health system. The timeframe will be the duration of the trial (62 weeks). All costs will be presented in Australian dollars for the current cost year 2016. Changes to health benefits will be estimated in quality-adjusted life years (QALYs). All costs and health benefits arising in future periods will be appropriately discounted [38].

#### Cost of implementing the bundle

Standard procedures for costing the intervention will be followed and all resources used to implement the intervention will be identified, measured and valued at local market prices [38]. The main resources used in implementing the bundle include staff time, costs of cleaning products, invisible gels, ultraviolet lamps and pathology costs. Resource use will be collected from each hospital by a monthly survey. Using a standardised tool across all hospitals and applying common cost vectors will ensure consistency in collection of costs across all sites and reduce uncertainty in estimates that often results from using retrospective administrative data. Staff time will be valued using full employment costs incurred at participating sites. Consumables will be valued at local market prices based on purchase contracts for each hospital.

#### Potential cost savings from implementing the bundle

If the bundle reduces infection rates, it should also produce cost savings. A reduced rate of infection should result in a lower average length of stay for patients and lower costs associated with diagnosis and treatment of infections [39]. Estimates of the number of antibiotics, screening and diagnostic tests and medical and surgical procedures avoided per infection will be based on standard treatment regimens for infection in participating hospitals and valued using hospital supplied unit costs. Estimates of the extra length of stay per infection avoided and the value of these bed days will be based on published estimates from recent high quality Australian studies [40, 41].

#### Change in health benefits from implementing the bundle

Changes to health benefits will be informed by estimating the deaths prevented, life years gained and QALYs gained as a result of fewer infections. The number of deaths averted will be estimated using Australian data on the attributable mortality associated with infection [42, 43]. We will also estimate years of life gained (accounting for observed age and comorbidities in the patient population), and finally, preference-based utility scores from the published literature [44] will be used to weight life expectancy, allowing the QALYs gained to be calculated.

#### Cost-effectiveness analysis

The incremental cost-effectiveness ratio (ICER) will be estimated by dividing the incremental change in total costs incurred and benefits gained due to implementing the bundle to give the incremental cost per QALY. Results will also be presented as the cost per infection prevented to allow comparison with other infection control interventions. The cleaning bundle will be considered cost-effective if the cost per QALY is less than $64,000, which is the willingness to pay for Australian QALYs and cost-saving if there is a net reduction in costs [45].

Probabilistic sensitivity analyses will be used to estimate the probability a decision to adopt the intervention is cost effective, given the current uncertainties in the observed parameters [46]. Sub-analyses will look at the efficiency of the cleaning bundle at individual hospitals to identify any systematic differences in the ICER that may be related to hospital characteristics.

### Ethics approval

This project has received ethics approval from the Uniting Care Health Human Research Ethics Committee (approval number 1413) and the Queensland University of Technology Human Research Ethics Committee (approval number 1400000828). Local ethics approvals are being completed for all participating hospital sites.

### Trial status

At the time of manuscript submission, the REACH study is beginning year 2. The study team is completing the recruitment of trial sites, multi-site ethical applications and agreements prior to randomisation and allocation.

## Discussion

### Limitations

This trial requires 11 participating hospitals. It could be difficult to recruit that number as it is very likely that some hospitals will decline or be unable to participate. This could be due to a number of reasons, e.g. disinterest in research, a lack of resources, a concern about negative impact on hospital budgets due to the need to implement change and increased product use associated with participation. This will be addressed by following an established recruitment process that provides clear information about what is required and a focus on fostering engagement and relationships with key decision makers at each site.

Once enrolled, willingness to participate may waiver for sites that are randomised to later trial commencement times. This will be addressed by maintaining communication with all sites and through ongoing monitoring of site context and engagement. The potential for fatigue related to participation, especially for the longer intervention phase sites, will be managed through frequent monitoring and engagement activities at the trial site.

We recognise some possible limitations in relation to the ‘opt-in’ nature of the recruitment process (i.e. that participating hospitals may be those most receptive to change and quality improvement). To mitigate this, we will as much as possible approach a range of eligible hospitals and will ensure we complete comprehensive context mapping at each site to fully understand the barriers and enablers that may have influenced the site’s decision to participate.

Other limitations could occur due to adverse events occurring at the staff, patient or hospital level during the trial. This could include outbreaks of HAIs, seasonal factors, organisational or policy changes. This will be mitigated by frequent monitoring of each site. Participating sites will agree to not implement changes that could impact the trial, e.g. cleaning policies or product use, without prior agreement from the study team. If there is a serious national epidemic during the trial the study will continue to collect data, but any planned changeovers to the intervention will be delayed until the epidemic has passed.

### Significance

Previous cleaning research has often focused on single interventions, such as a new cleaning product or auditing strategy. However, implementing cleaning in a hospital setting is a multifaceted and complex process, where contextual factors influence the success of a new approach. This study, with its implementation science approach and emphasis on facilitation and local hospital context, will enable us to develop new insights into the processes and impacts of hospital cleaning. These will be used to assist with the translation of new knowledge from each of the trial sites to other hospitals in Australia and internationally.

The effectiveness and cost-effectiveness of a cleaning bundle intervention will be evaluated to support evidence-based decision-making in public and private healthcare sectors about the impact of investment in an environmental cleaning programme in acute hospitals, particularly on the risks of HAIs. It will further show whether this policy is cost-effective in a real world setting, addressing an identified research gap [7].

## Abbreviations

ACSQHC: Australian Commission on Safety and Quality in Health Care
ANZCTR: Australia New Zealand Clinical Trial Registry
ATP: adenosine tri-phosphate
AUD: Australian dollar
CDI: *Clostridium difficile* infection
CRE-RHAI: Centre of Research Excellence in Reducing Healthcare-Associated Infections
GLMM: generalised linear mixed model
HAI: healthcare-associated infection
ICER: incremental cost-effectiveness ratio
ICU: intensive care unit
iPARIHS: integrated Promoting Action on Research Implementation in Health Services
MRSA: methicillin-resistant *Staphylococcus aureus*
NHMRC: National Health and Medical Research Council
NHPA: National Health Performance Authority
QALY: quality-adjusted life years
REACH: Researching Effective Approaches to Cleaning in Hospitals
RLU: relative light unit
SAB: *Staphylococcus aureus* bacteraemia
UV: ultraviolet
VRE: vancomycin resistant enterococci
